# IDF-Net: Interpretable Dynamic Fusion Network for Colorectal Cancer Diagnosis Using Cross-Modal Imaging

**DOI:** 10.3390/diagnostics16010099

**Published:** 2025-12-27

**Authors:** Helen Haile Hayeso, Peifeng Shi, Jingwen Lian, Zenebe Markos Lonseko, Nini Rao

**Affiliations:** Department of Biomedical Engineering, University of Electronic Science and Technology of China, Chengdu 610054, China; helenh@std.uestc.edu.cn (H.H.H.); 202421140102@std.uestc.edu.cn (P.S.); lianjingwen_xoxo@163.com (J.L.); zenebe@uestc.edu.cn (Z.M.L.)

**Keywords:** colorectal cancer diagnosis, gastrointestinal disease, dynamic fusion network, deep learning, machine learning, cross-modal imaging, interpretable AI

## Abstract

**Background/Objectives**: Colorectal cancer (CRC) is a leading cause of cancer deaths worldwide, underscoring the need for diagnostic tools that early, accurate, and clinically interpretable. Current artificial intelligence (AI) models are predominantly unimodal and lack sufficient interpretability, which restricts their clinical adoption. **Methods**: We propose IDF-Net, an interpretable dynamic fusion framework that integrates endoscopy, computed tomography (CT), and histopathology using modality-specific encoders, a dual-stage adaptive gating mechanism, and cross-modal attention. We conducted stratified 5-fold cross-validation and assessed interpretability using spatial heatmaps and modality attribution. We also quantified the results using the intersection-over-union metric for saliency alignment. **Results**: IDF-Net achieved a state-of-the-art accuracy of 0.920 (0.907–0.936) and area under the curve (AUC) of 0.991 (95% CI: 0.965–0.997), significantly outperforming unimodal and static-fusion baselines (*p* < 0.05). Interpretability analysis of IDF-Net demonstrated a strong alignment between Gradient-weighted Class Activation Mapping++ heatmaps and expert-annotated lesions, as well as case-specific modality contributions via SHapley Additive exPlanations values. Ablation studies confirmed the contribution of each component, with dynamic routing and cross-attention fusion improving AUC by 0.038 and 0.046, respectively. **Conclusions**: IDF-Net introduces a dynamically fused, multimodal diagnostic framework with integrated quantitative interpretability, demonstrating superior accuracy and strong potential for clinical translation in CRC diagnosis. The model’s adaptive design allows it to function robustly even when CT data is unavailable, aligning with common clinical pathways while leveraging additional imaging when present for comprehensive staging.

## 1. Introduction

Among gastrointestinal diseases (GIDs), colorectal cancer (CRC) poses a particularly formidable global health challenge, ranking as the third most diagnosed cancer and the second leading cause of cancer-related mortality worldwide [1,2]. Recent studies indicate approximately 1.9 million new cases and 935,000 deaths annually, with projections suggesting a dramatic rise in incidence by 2040 [1,2,3]. This growing burden, coupled with substantial economic costs, underscores an urgent need for enhanced diagnostic strategies that improve early detection, accurate staging, and severity estimation [4,5]. The current diagnostic paradigm depends on a multimodal diagnosis, including endoscopy for direct visualization, computed tomography (CT) for staging, and histopathology for definitive confirmation. However, these modalities are typically interpreted in isolation, resulting in fragmented assessment, potential diagnostic delays, and limited integration of complementary cross-modal information essential for accurate CRC evaluation [4,6].

Artificial intelligence (AI), particularly deep learning (DL), has emerged as a transformative force in gastrointestinal (GI) diagnostics, demonstrating remarkable performance in tasks such as lesion detection, classification, and segmentation [7,8]. For example, AI-assisted endoscopy systems have shown improved adenoma detection rates, and DL models can accurately classify cancerous regions in histopathology whole-slide images [9,10]. Despite substantial progress, current AI systems face two key limitations hindering clinical adoption: (1) the predominance of unimodal models that overlook the inherently multimodal nature of CRC diagnosis [11], and (2) insufficient interpretability, which limits clinical trust and hinders integration into routine procedures [12].

Furthermore, existing multimodal approaches typically use static fusion with fixed modality weights that cannot adjust to patient-specific image quality or diagnostic context [13,14]. A recent study [14] has pioneered dynamic fusion, which often relies on single-stage weighting mechanisms that can be prone to modality collapse and lack explicit modeling of inter-modality relationships. This limitation fails to accurately reflect real-world clinical decision-making, where the significance of each modality varies on a case-by-case basis. These gaps highlight the need for a dynamic, interpretable fusion framework that can integrate complementary signals from mucosal, structural, and cellular imaging domains.

To address these challenges, we propose IDF-Net, an interpretable DL framework that dynamically fuses endoscopy, CT, and histopathology using a dual-stage gating mechanism and cross-modal attention. IDF-Net is proposed to mirror clinical reasoning and adaptively weight modalities based on patient-specific diagnostic relevance.

The main contributions of this work are summarized below:

1. We propose IDF-Net, a multimodal fusion framework that adaptively integrates endoscopy, CT, and histopathology through dual-stage gating and cross-modal attention, addressing the limitations of static or unimodal diagnostic models.
2. We design a clinically consistent multimodal benchmark using diagnosis-matched pairing from large-scale public datasets, enabling integrated analysis of mucosal, structural, and cellular features for CRC.
3. IDF-Net incorporates gradient-weighted class activation mapping++ (Grad-CAM++) lesion localization and SHapley Additive exPlanations (SHAP)-based modality attribution, achieving strong alignment with expert annotations and providing transparent, clinically meaningful explanations.
4. IDF-Net achieves superior accuracy across both internal and external validation sets, and remains robust in scenarios with missing modalities, demonstrating its readiness for real-world clinical workflows.

## 2. Materials and Methods

### 2.1. Datasets and Study Design

**Figure 1:**
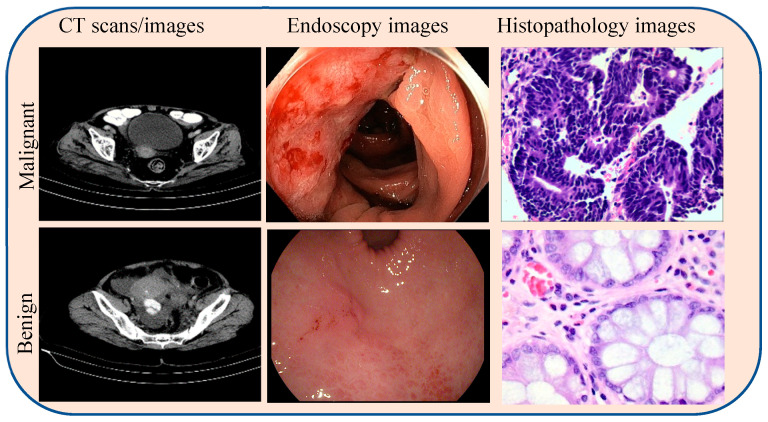
Example of multimodal data for CRC diagnosis. Representative images for a benign case (**bottom row**) and a malignant case (**top row**): contrast-enhanced CT scan (**left**), endoscopic image (**center**), and histopathology slide (**right**).

This study utilized publicly available endoscopy, CT, and histopathology datasets to construct a trimodal resource for colorectal cancer (CRC) diagnosis. Histopathology images were obtained from The Cancer Genome Atlas Colon Adenocarcinoma (TCGA)-Cancer Genome Atlas Colon Adenocarcinoma (COAD) (TCGA-COAD) (8387 images) [15] and the LC25000 dataset (10,000 images) [16]. Endoscopic images were sourced from HyperKvasir [17], Kvasir-SEG [18], and GastroVision [19], yielding approximately 20,000 high-quality colonoscopy frames after quality filtering. CT images were collected from TCGA-COAD (8387 images) and ColonCancerCT-2025 (11,340 images) [20]. The datasets were split into training (70%), validation (15%), and testing (15%) sets. Sample images are presented in Figure 1.

Since naturally paired trimodal datasets are rarely publicly available, we employed diagnosis-matched pairing to construct clinically consistent trimodal samples. Labels were harmonized using pathology-confirmed TCGA diagnoses as the reference standard, and sensitivity analyses were performed to minimize bias from cross-dataset variability.

### 2.2. Data Preprocessing

All imaging modalities underwent standardized preprocessing to ensure consistency and optimize multimodal learning. Endoscopic images (resolution: 720 × 576 to 1920 × 1080) were extracted, anonymized, and resized to 512 × 512 pixels. Pixel intensities were normalized to have a zero mean and unit variance, followed by data augmentation using random rotations (±15°), horizontal and vertical flips, brightness and contrast adjustments, and a Gaussian blur (σ = 0.5) to enhance robustness under real-world variability. CT images were resampled to an in-plane resolution of 1.0 mm × 1.0 mm and linearly normalized to the [0, 1] range after clipping Hounsfield-unit intensities to [−150, 350]. Given the practical constraint of working with slice-based data from public repositories, a pretrained 2D U-Net model was selected for colorectal region segmentation. Tumor-centered slices were cropped to 128 × 128 pixels to focus the analysis on diagnostically relevant regions (RoI). Identification was performed slice-by-slice, and an expert radiologist manually reviewed all outputs to ensure consistency. While this 2D approach is a simplification of the 3D volume, it provides a computationally efficient and data-compatible method for region-of-interest extraction, and the subsequent dynamic gating mechanism provides robustness to potential segmentation inaccuracies. Histopathology images scanned at 40× magnification was used to perform sensitivity analyses on non-overlapping 512 × 512-pixel patches at 0.25 µm/pixel. Patches covering more than 50% of the tissue area were retained, and stain normalization using the Reinhard method was applied to mitigate inter-slide color variability. All datasets were harmonized to a binary CRC label (benign vs. malignant). To ensure rigorous evaluation and avoid patient-level leakage, we used a stratified five-fold cross-validation, confirming that no patient’s data appeared in any fold.

To enhance generalization, data augmentation included random rotations (±20°), horizontal/vertical flips, Gaussian noise, and contrast jittering (±10%). Random Weighted Sampling (RWS) was employed to alleviate class imbalance by assigning sampling probabilities inversely proportional to class frequencies [21], where the sampling weight $W_{s}\left(i\right)$ for category i was set inversely proportional to its sample size $N_{s}\left(i\right)$ expressed in equations (Equations (1)–(3)).

(1) $$W_{s}\left(i\right)=\frac{1}{N_{s}\left(i\right)},N_{s}\left(i\right)>0,i=1,2,\ldots N$$

Furthermore, data normalization ${(X}_{norm}$) and standardization (Z-score) are key preprocessing steps for better performance of algorithms and expressed in Equations (2) and (3).

(2) $$X_{norm}(x^{\prime })=\frac{x-min(x)}{max(x)-min(x)}$$

where $x^{\prime }$ is the normalized value of feature x, and min(x) and max(x) are the minimum and maximum values of feature x across the dataset.

(3) $$X_{std}=(x^{\prime })=x-\mu x∕\sigma x$$

where $\mu_{x}$ is the mean and $\sigma_{x}$ is the standard deviation (SD) of feature x. $X_{norm}$ is used when we aim for a bounded data [0, 1] scale, while $X_{std}$ is preferable when the data has Gaussian-like properties.

### 2.3. IDF-Net Architecture

**Figure 2:**
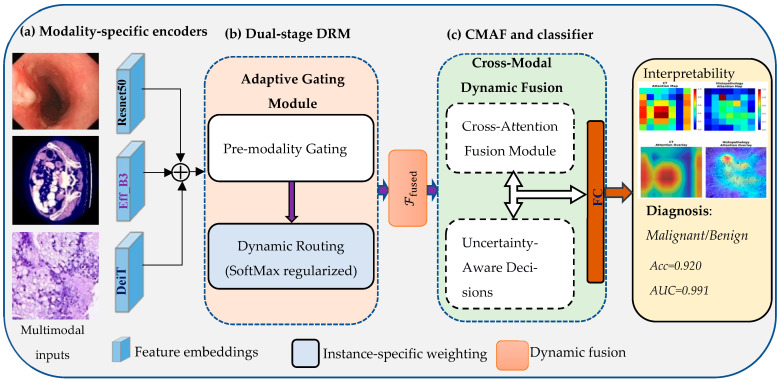
Illustrates the overall architecture of the interpretable dynamic fusion network. (**a**) Modality-specific encoders extract latent features from endoscopy, CT, and histopathology images. (**b**) A DRM applies pre-modality gating and SoftMax-based routing with entropy regularization to generate adaptive modality weights. (**c**) Cross-modal attention fuses routed embeddings via a multi-head QKV mechanism with uncertainty-aware decisions, followed by a FC classifier and integrated interpretability.

The proposed framework integrates multimodal medical images: endoscopic, CT, and histopathology data through a hierarchical, interpretable DL framework. Figure 2 illustrates an overall framework of the proposed IDF-Net, which consists of three sequential modules: (a) modality-specific encoders, (b) a dual-stage dynamic routing module (DRM), and (c) a cross-modal attention fusion (CMAF) and classification and interpretability head. In the first stage, each imaging modality (endoscopy, CT, and histopathology) is processed independently using a modality-optimized encoder (ResNet-50, EfficientNet-B3, and DeiT (ViT-Base), respectively). These encoders extract latent feature embeddings representing surface-level mucosal patterns, structural radiologic cues, and cellular-level morphologic characteristics. The second panel demonstrates the dual-stage DRM, which first applies pre-modality gating to compute a case-specific reliability coefficient for each modality. These coefficients attenuate noisy or low-quality embeddings before they are passed to a SoftMax-based routing layer. An entropy-regularization term prevents the model from collapsing onto a single modality, ensuring balanced and adaptive weighting across endoscopy, CT, and histopathology. The output of this stage is a set of routed embeddings with learned modality importance. Finally, third panel depicts the cross-modal dynamic fusion module, where the routed embeddings are integrated using a multi-head query–key–value (QKV) attention mechanism. This allows each modality to attend to and refine complementary information from the others, generating a unified 2048-dimensional representation. This fused vector is passed through a fully connected (FC) classifier to produce the malignancy probability. Integrated explainability mechanisms generate spatial saliency maps and modality-level contribution scores, enabling case-specific interpretability and supporting clinical review.

Endoscopy, CT, and histopathology images are processed by modality-specific encoders (ResNet-50, EfficientNet-B3, and ViT-Base, respectively) to produce latent embeddings $f_{e},\;f_{c},\;f_{h}$. A dual-stage approach first applies pre-modality gates to estimate case-specific reliability scores and then utilizes a SoftMax-based routing layer with entropy regularization to obtain normalized modality weights $g_{i}$. The gated embeddings are passed to a multi-head cross-modal attention block that performs QKV fusion across modalities, generating a unified 2048-dimensional representation. This fused vector is fed into a FC classifier that outputs the probability of malignancy. Grad-CAM++ and SHAP provide spatial and modality-level explanations, enabling case-specific interpretability of IDF-Net’s predictions.

#### 2.3.1. Modality-Specific Feature Encoders

Each imaging modality is processed through an encoder tailored to its spatial and textural characteristics:

- Endoscopic Encoder: A modified ResNet-50 pretrained on ImageNet was used to extract discriminative features from 512 × 512 RGB endoscopic frames. The final convolutional block outputs a 1024-dimensional embedding representing mucosal textures, vascular structures, and lesion morphology.
- CT Encoder (2D): As the CT dataset consists of 2D axial slices, a convolutional neural network (CNN) adapted from the EfficientNet-B3 architecture was employed. Each slice was resized to 512 × 512 pixels, normalized to [0, 1], and intensity-clipped to [−150, 350] Hounsfield units. The encoder generates a 1024-dimensional latent vector that extracts radiologic features, such as density variations, wall thickening, and tumor boundaries, within the colorectal region. Global average pooling aggregates slice-level features into a single case-level embedding. This 2D approach was selected to maintain computational efficiency and compatibility with datasets lacking complete 3D volume information.
- Histopathology Encoder: A DeiT (ViT-Base) model pretrained on a large-scale histology corpus was used to capture cell-level and glandular features from H&E-stained patches (512 × 512). Attention pooling aggregates patch embeddings into a case-level 1024-dimensional representation, enabling fine-grained learning of tumor architecture and cytological patterns.

All three encoders thus project their inputs into a shared 1024-dimensional latent space, enabling subsequent cross-modal interaction.

#### 2.3.2. Adaptive Gating Mechanism

The adaptive gating mechanism consists of a dual-stage design. *Stage 1: Pre-modality Reliability Gating:* To attenuate unreliable or low-quality modalities, each feature embedding $f_{m}$ first passes through a learnable sigmoid gate, producing a reliability coefficient $g_{m}$ (Equation (4)) and a gated embedding. $f_{m}=g_{m}\cdot f_{m}$. *Stage 2: Entropy-Regularized Dynamic Routing*: The gated embeddings as ${\tilde{f}}_{m}$ are then processed by the dynamic routing module. This module computes the final, normalized fusion weights $g_{i}$ using a SoftMax function. An entropy-regularization term ($L_{ent}=-\lambda \sum_{i}g_{i}\log{(g}_{i}))$ is added to the overall loss (Section 2.4). The hyperparameter λ was empirically set to 0.01 to stabilize training. This promotes balanced, case-adaptive fusion. The coefficient λ was empirically tuned to ensure stable gradient flow and prevent dominance of any single modality.

The adaptive gating mechanism operates in two distinct, sequential stages to ensure robust and balanced fusion, as illustrated in Figure 2b. The process is as follows. (1) The DRM produces gating weights regularized by an entropy loss, explicitly preventing the model from collapsing its reliance onto a single modality and enforcing a more balanced, robust use of all inputs. (2) In conventional gating, modalities are weighted, but their features do not deeply interact; in contrast, our CAF uses a bidirectional multi-head attention mechanism to allow features from each modality to attend to and refine those of the others. This enables IDF-Net to move beyond simple weighting toward actual integrative reasoning.

To enhance robustness across variable data quality, a learnable gating mechanism first computes a per-case weighting coefficient $g_{m}$ for each modality embedding $f_{m}$. The gated embedding for each modality is calculated as ${\tilde{f}}_{m}=$ $g_{m}\cdot f_{m}$. This step attenuates unreliable or missing modalities before fusion. For each modality m∈{E,C,H} (Endoscopy, CT, Histopathology), the gating coefficient $g_{m}$ is computed as

(4) $$g_{m}=\sigma (w_{m}f_{m}+b_{m})$$

where $f_{m}$ represents the modality embedding, $w_{m}$ and $b_{m}$ are learnable parameters, and σ(⋅) is the Sigmoid function, g for gate, and w for the weight matrix. The gated embeddings are aggregated as described in Equation (5).

(5) $$F_{fused}=\sum_{m}g_{m}{\cdot f}_{m}$$

where $g_{m}$ is the gating coefficient from Equation (4). This mechanism allows IDF-Net to attenuate unreliable or missing modalities while emphasizing those of higher diagnostic confidence, improving adaptability in real-world clinical workflows. This dynamic weighting ensures that no single modality is pre-defined as dominant. Instead, the model learns to assign higher influence to the most reliable and informative features for each case. An entropy regularization term within the routing module explicitly prevents the model from collapsing its reliance onto a single modality (e.g., histopathology) unless strongly justified by the input data, ensuring a balanced, context-aware fusion that mirrors nuanced clinical judgment.

The feature embeddings are passed to the DRM, which computes adaptive gating weights gᵢ ∈[0,1] to modulate the contribution of each modality. This mechanism allows the model to emphasize the most significant inputs and attenuate noisy ones, effectively simulating a prioritization of data quality. These embeddings are routed to the DRM, which computes adaptive gating weights $(g_{1}$, $g_{2}$, $g_{3}$) in a data-driven manner, enabling the model to emphasize the most informative modality while attenuating noisy or incomplete inputs [22]. The gating coefficients are computed via a SoftMax-based routing function expressed in Equation (6):

(6) $$gᵢ=\frac{exp(W_{i}\cdot f_{i}+b_{i})}{\sum_{j=1}^{3}exp(W_{j}\cdot f_{j}+b_{j})}$$

where $f_{i}$ represents the intermediate feature map of the *i*th modality, $W_{i}$ and $b_{i}$ are learnable parameters defining the routing gate, and gᵢ dynamically scales the modality contribution during fusion.

#### 2.3.3. Cross-Modal Dynamic Fusion

The gated embeddings ${\tilde{f}}_{E},\;{\tilde{f}}_{C},\;{\tilde{f}}_{H}$ are then passed through a multi-head cross-attention module to model contextual relationships between modalities. The resulting fused representation serves as a holistic diagnostic descriptor encompassing surface, structural, and cellular information.

The gated embeddings {${\tilde{f}}_{E},\;{\tilde{f}}_{C},\;{\tilde{f}}_{H}$} are concatenated into token sequence and refined via a multi-head cross-attention fusion mechanism (CAF) module with 8 attention heads. Within this module, linear projection layers generate query (Q), key (K), and value (V) matrices from the combined sequence, allowing each modality to attend to and be attended by all others in a fully connected manner. This design enables deep, bidirectional feature interaction within a shared 1024-dimensional latent space, moving beyond simple weighting toward integrative reasoning.

#### 2.3.4. The Classification and Interpretability Head

The fused representation is passed through a two-layer FC classifier (2048→512→1) with ReLU activation, dropout regularization, and a sigmoid output, yielding the malignancy probability. To improve transparency, IDF-Net integrates dual interpretability mechanisms:

▪ Grad-CAM++ is utilized to highlight spatially salient regions within each encoder, visualizing class-discriminative areas in endoscopic and histopathologic inputs.
▪ SHAP analysis quantifies the contribution of each modality and fused feature to the final prediction, supporting clinician-oriented interpretability.

### 2.4. Model Training Loss Functions

The proposed model was trained using a composite loss function designed to optimize both classification accuracy and stable multimodal fusion. The primary objective function was the binary cross-entropy (BCE) loss, which quantified the discrepancy between the predicted malignancy probabilities and the ground truth labels. To stabilize dynamic gating, an entropy-regularization term was added to prevent modality collapse and encourage balanced contributions from all modalities during training. This entropy term penalized overly confident gating distributions and promoted adaptive, context-dependent weighting across endoscopy, CT, and histopathology embeddings. For interpretability consistency, a mild L2 weight regularization was applied across encoder and fusion parameters to reduce overfitting and improve generalization to unseen institutions. The final training loss was expressed as a weighted sum: ${L_{total}=L}_{BCE}+{\lambda L}_{ent}+{\beta L}_{L2}$, where $L_{BCE}$ is the binary-entropy loss, $L_{ent}$ is the entropy regularization on routing weights $g_{i}$ and $L_{L2}$ is the weight decay. The coefficient λ was empirically set to 0.01 and β=0.001. This composite objective optimizes classification accuracy while ensuring stable and balanced multimodal fusion.

### 2.5. Training Implementation Details

IDF-Net was implemented in Python 3.10 using PyTorch 2.5. All experiments were conducted on an NVIDIA A100 GPU (80 GB). Training used AdamW (learning rate = 1 × 10^−4^, weight decay = 0.01, β_1_ = 0.9, β_2_ = 0.999), batch size = 16, and a cosine annealing learning-rate schedule (min LR = 1 × 10^−6^). Dropout (*p* = 0.3) was applied to the classifier layer. Early stopping (patience = 10 epochs) was monitored based on the validation AUC. To enhance reproducibility, we fixed all random seeds (Torch/NumPy/Python) to 42.

### 2.6. Evaluation Metrics

Model performance was assessed using standard evaluation metrics, including accuracy, area under the receiver operating characteristic curve (AUC), precision, recall, and F1-score as expressed in Equations (7)–(10). Statistical significance of performance differences was evaluated based on (p<0.05), and 95% confidence intervals (CI) were estimated via bootstrap resampling (*n* = 1000). The spatial alignment of Grad-CAM heatmaps visualization with expert-annotated lesion masks was quantitatively evaluated using the intersection over union (IoU) metric. Calibration was assessed via calibration curves and Brier score. Decision curve analysis (DCA) was performed to evaluate net clinical benefit. The McNemar test was used to assess the statistical significance of performance differences between IDF-Net and baseline models. A significant difference (*p* < 0.05) indicated that the improvement in diagnostic accuracy was unlikely to have occurred by chance.

(7) $$Precision=\frac{TP}{TP+FP},$$

(8) $$Recall=\frac{TP}{TP+FN},$$

(9) $$Accuracy=\frac{TP+TN}{TP+FP+TN+FN},$$

(10) $$F1\;score=\frac{2\times \left(Precision\times Sensitivity\right)}{Precision+Sensitivity},$$

To comprehensively evaluate the diagnostic performance of IDF-Net and comparative baseline models, multiple quantitative metrics were calculated based on the confusion matrix, including accuracy, sensitivity (recall), specificity, precision, F1-score, and the area under the receiver operating characteristic curve (AUC). Accuracy was computed as the overall proportion of correctly classified cases, whereas sensitivity measured the ability to identify malignant colorectal lesions. The F1-score provided a balanced harmonic mean of precision and recall, thereby mitigating the effects of class imbalance. The AUC provided a threshold-independent measure of discrimination, reflecting the probability that the model ranks a randomly selected malignant sample higher than a benign one.

### 2.7. Statistical Analysis

Model performance was evaluated using accuracy, precision, recall, F1-score, and the area under the ROC curve (AUC-ROC). CI (95% CI) for AUC were computed using 1000-sample bootstrap resampling. Differences in classification accuracy between IDF-Net and unimodal models were evaluated using McNemar’s test, which is appropriate for paired binary outcomes. Differences in AUC were assessed using DeLong’s test, the standard nonparametric method for comparing correlated ROC curves. Model calibration was evaluated using the Brier score and reliability curves. DCA quantified the net clinical benefit across threshold probabilities (0.05–0.50). Interpretability heatmap alignment was quantified using Intersection over Union (IoU) against expert annotations.

All experimental code and preprocessing pipelines will be made publicly available upon acceptance.

## 3. Results

### 3.1. Overall Performance

IDF-Net achieved an accuracy of 0.920 and F1 score of 0.918, demonstrating a substantial improvement over all unimodal and static-fusion baselines. The model’s mean AUC across the validation folds was 0.991 (95% CI: 0.965–0.997) as shown in Table 1 and Table 2. This reflects excellent discriminative capacity: the model will rank a randomly chosen malignant case above a randomly chosen benign case in more than 95% of pairwise comparisons. Importantly, performance was consistently high across all five folds, suggesting that the model generalizes well to different subsets of the multi-institutional data.

**Figure 3:**
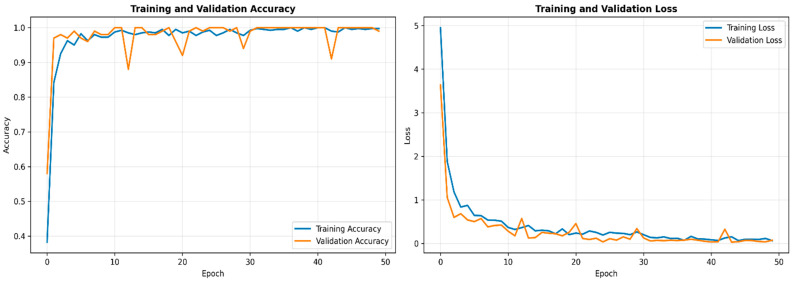
Training and validation performance curve of the IDF-Net. Training and validation accuracy and loss curves for IDF-Net over 100 epochs, demonstrating stable convergence.

Figure 3 shows the training and validation accuracy and loss trajectories of IDF-Net across epochs, demonstrating stable convergence without overfitting. Training and validation curves remain closely aligned, indicating effective regularization and strong generalization of the trimodal fusion framework.

### 3.2. Comparative Analysis with SOTA Methods

To contextualize IDF-Net’s diagnostic performance, we compared it with recent state-of-the-art multimodal and unimodal AI systems (Table 1). Although direct comparison is limited by dataset and task differences, several observations emerge.

IDF-Net achieved the highest performance among all compared models (AUC = 0.991), outperforming unimodal baselines and static-fusion multimodal frameworks. These improvements validate the advantage of dynamic gating and cross-modal attention mechanisms. For example, Wang et al. [23] achieved an AUC of 0.988 in patch-level histopathology classification; however, their approach is unimodal and does not incorporate radiologic or endoscopic information, which is essential for comprehensive CRC assessment. Similarly, DeepFuse-CRC [3] uses early feature concatenation, which lacks the flexibility to adapt to case-specific variations in data quality. More recent multimodal architectures, including M3F-Net [10] and GAIT [13], highlight emerging interest in cross-modal fusion. However, these models primarily rely on static fusion strategies, which employ uniform weighting across all patients.

In contrast, IDF-Net’s dynamic gating mechanism adjusts fusion weights on a per-case basis, better reflecting real-world heterogeneity. The Multi-IOMM framework [24] also targets GI cancers but integrates only endoscopy and histopathology, limiting its relevance for staging. IDF-Net’s multimodal integration more closely mirrors clinical workflows.

Finally, the proposed model provides quantitative interpretability via SHAP-based attribution alongside Grad-CAM++ visualizations, offering case-specific. This integration of dynamic fusion and transparent explainability strengthens IDF-Net’s potential for clinical adoption.

### 3.3. Addressing Missing Modalities

To evaluate robustness under incomplete inputs, a common clinical scenario, we conducted a missing-modality analysis. A modality was simulated as “missing” by setting its original input image to a zero tensor before processing by its encoder. The model’s adaptive gating and fusion mechanisms then had to operate on the resulting non-informative embedding. As expected, removing any modality reduced performance relative to the complete trimodal design. As expected, removing any modality reduced performance relative to the complete trimodal design. Excluding histopathology produced the most significant decline (AUC = ~0.93), consistent with its strong individual discriminatory power. Removing endoscopy or CT resulted in a moderate decrease. Despite these reductions, all bimodal variants still outperformed unimodal models, demonstrating that IDF-Net effectively leverages remaining modalities and maintains resilience to incomplete clinical data, a common scenario in real-world workflows.

### 3.4. Statistical Comparison of Model Performance

The model’s mean AUC was 0.991 (95% CI: 0.965–0.997) across the validation folds. McNemar’s test revealed significant improvements in classification accuracy compared to each unimodal model (*p* < 0.01), confirming that the performance gains were not due to random variation. The added complexity of dynamic fusion incurs a modest computational overhead. On an NVIDIA A100 graphical processing unit (GPU), the average inference time for the full trimodal IDF-Net was approximately 120 ms per case, compared to 35–40 ms for a unimodal endoscopic model and 75–85 ms for a bimodal model. This latency remains well within acceptable limits for a non-real-time decision support tool and demonstrates a favorable trade-off between performance gain and computational cost. Together, these findings demonstrate that dynamic gating and cross-modal fusion enable IDF-Net to better exploit complementary multimodal information, resulting in enhanced sensitivity and specificity for CRC diagnosis.

This consistent performance across folds underscores the stability and generalizability of IDF-Net under variable imaging conditions. Thus, these findings confirm that trimodal fusion substantially advances diagnostic performance compared with unimodal baselines. In particular, IDF-Net offers significantly higher sensitivity and specificity, indicating improved discrimination of malignant lesions across modalities.

### 3.5. External Validation and Generalizability

External validation was performed using independent endoscopy, CT, and histopathology datasets representing diverse institutions and imaging conditions. IDF-Net experimentally demonstrated consistently high performance across all external datasets (Table 3), with AUC values exceeding 0.91 for every modality. These results show that the adaptive gating mechanism and cross-modal attention fusion modules generalize effectively to unseen data sources, despite variations in device type, imaging quality, and institutional characteristics. The stability of performance across modalities, mucosal (endoscopy), structural (CT), and cellular (histopathology), suggests that IDF-Net learns robust multimodal representations that transfer well without modality-specific fine-tuning.

Collectively, these results support the potential of the proposed model as a clinically deployable, multimodal diagnostic framework suitable for real-world GI imaging settings.

### 3.6. Ablation Study

To determine the contribution of each architectural component, we performed an ablation study by incrementally adding the DRM, CAF, and the interpretability module to a baseline CNN without fusion.

As summarized in Table 4, each component produced measurable performance gains. Incorporating the DRM improved discrimination (ΔAUC = +0.038) by enabling adaptive modality weighting. Adding the CAF further enhanced multimodal feature integration (ΔAUC = +0.046). The full IDF-Net, which combines DRM, CAF, and IM, achieved the highest performance (AUC = 0.991 ± 0.012), confirming that each module contributes to accuracy, robustness, and interpretability.

Table 5 presents the performance of the proposed model under missing-modality conditions.

**Figure 4:**
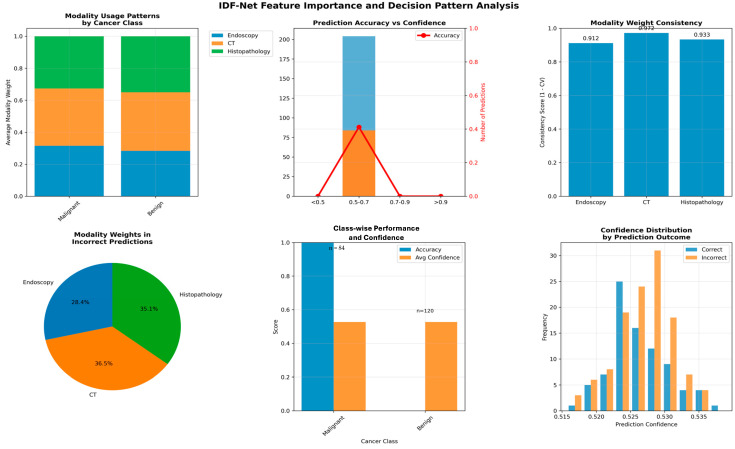
Interpretability of feature importance and decision pattern analysis of IDF-Net.

Figure 4 reveals that IDF-Net produces clinically aligned and modality-aware interpretability, with Grad-CAM++ heatmaps accurately localizing lesion-specific regions and SHAP analysis quantifying modality contributions on a per-case basis. Compared with unimodal and static-fusion baselines, IDF-Net demonstrates tighter overlap between attended regions and expert-annotated tumor boundaries, reflecting a more realistic mapping of diagnostic cues. The model consistently emphasizes mucosal patterns in endoscopy, structural abnormalities in CT, and cellular atypia in histopathology when these modalities are most informative, indicating that the dynamic gating mechanism effectively selects context-relevant features. Overall, IDF-Net not only advances performance but also provides transparent, case-specific interpretability, making it suitable for clinical decision support.

## 4. Discussion

This study presents IDF-Net, a multimodal interpretable fusion framework that integrates endoscopy, CT, and histopathology to improve CRC diagnosis. The model substantially outperformed unimodal and static fusion baselines, achieving an AUC of 0.991 with balanced sensitivity and specificity.

### 4.1. Novelty and Strengths

IDF-Net advances prior work by introducing a dynamic, clinically aligned multimodal fusion framework that combines mucosal, structural, and cellular information. Unlike static fusion [23], the dual-stage gating and cross-modal attention modules enable adaptive, case-specific interaction among modalities, better approximating the reasoning steps used by clinicians. Second, our work enhances the state of the art in adaptive fusion. Unlike single-stage gating in models like AdaFuse [25] and DynMM [22], which compute a single set of weights, IDF-Net employs a dual-stage gating mechanism. The first stage (pre-modality gating) acts as a reliability filter, attenuating noisy inputs before they are fused. The second stage (entropy-regularized routing) explicitly prevents modality collapse by penalizing overconfident distributions, ensuring a balanced, context-aware use of all three modalities. Furthermore, while prior methods often perform shallow weighting, our cross-modal attention module enables deep, bidirectional feature interaction, allowing modalities to refine each other’s representations, a key step toward integrative reasoning that mirrors clinical intuition. Third, IDF-Net incorporates interpretability, combining Grad-CAM++ spatial heatmaps and SHAP-based modality attribution. Interpretability analysis demonstrated strong agreement between Grad-CAM++ localizations and expert-annotated lesions (mean IoU ≈ 0.65), indicating that IDF-Net consistently attends to clinically relevant regions. SHAP-based modality attribution further provided patient-specific transparency, supporting clinical interpretability [24]. This quantitative assessment distinguishes IDF-Net from models offering only post hoc qualitative visualizations. Fourth, by training and validating IDF-Net on trimodal patient profiles sourced from multiple institutions, we demonstrate strong domain generalization. Despite the inherent challenge of cross-dataset multimodal pairing, rigorous label harmonization and patient-level cross-validation ensured that trimodal fusion did not introduce leakage or bias. The consistent external validation performance emphasizes the robustness and adaptability of the proposed framework.

### 4.2. Implications for Clinical Practice

IDF-Net’s strong performance and interpretability suggest meaningful applications in clinical workflows. For endoscopists, real-time heatmaps (with a processing latency of <100 ms per frame) can highlight subtle mucosal lesions that would otherwise be overlooked, providing immediate visual guidance during colonoscopy. For radiologists, modality attributions can clarify whether CT features strongly influenced malignancy predictions. For pathologists, cell-level saliency can support more targeted slide review by directing attention to the most suspicious patches within a whole-slide image, potentially improving diagnostic efficiency. This multimodal synergy may reduce diagnostic delays and improve early cancer detection, particularly in resource-constrained settings. Importantly, while IDF-Net is designed to integrate CT for staging information where available, its architecture remains robust in bimodal (endoscopy + histopathology) settings, which represent a common diagnostic pathway. This flexibility ensures broader applicability across different clinical scenarios, from initial screening to comprehensive staging workups. Improved model sensitivity and specificity directly correlate with higher detection rates of clinically significant lesions (e.g., adenomas, early cancers), while high specificity may help decrease unnecessary biopsies. For example, the model’s high sensitivity means it can detect malignant lesions that might be missed if only one modality were considered, potentially enabling earlier intervention and improved patient outcomes. At the same time, its high specificity implies fewer false alarms, potentially reducing unnecessary follow-up procedures or biopsies. By alerting endoscopists to subtle lesions during colonoscopies, triaging CT scans for radiologists, and flagging pathology slides for a second look, IDF-Net could streamline the diagnostic workflow. Moreover, the model’s interpretability can enhance clinician trust. For instance, a Grad-CAM++ heatmap highlighting the specific polyp that led to a positive prediction or a SHAP plot indicating that histopathology was the dominant contributor in a case can help physicians validate and understand the AI’s reasoning. This transparency facilitates the integration of the system into clinical workflows. In the context of GI diseases, where the global burden remains high, and healthcare resources are finite, tools like IDF-Net may contribute to earlier detection, more accurate staging, and overall cost-efficient care by integrating information that is traditionally siloed.

The robustness and interpretability of IDF-Net position it as a valuable decision-support tool in a clinical setting. In a typical workflow, after a patient undergoes a colonoscopy, CT scan, and biopsy, IDF-Net can pre-integrate the three data sources to provide a unified malignancy risk score, along with spatial heatmaps and modality contribution plots. This would enable clinicians to quickly confirm the AI’s reasoning, focus their discussion on cases with discrepant findings across modalities or high AI confidence, and ultimately arrive at a more efficient, data-informed consensus diagnosis. The modular architecture of IDF-Net makes it compatible with real-time endoscopic platforms and PACS-integrated radiology workflows, enhancing its translational potential. This could streamline IDF-Net workflows, reduce inter-observer variability, and improve diagnostic confidence, particularly for challenging cases.

### 4.3. Clinical Rationale Integration

The clinical diagnosis of CRC inherently relies on a multimodal workflow, which includes endoscopy for direct visualization of the mucosa, CT for staging and detection of distant metastases, and histopathology for cellular confirmation. While each modality provides unique and complementary information, they are often interpreted in isolation from one another. An AI system that can dynamically fuse this information, mirroring the ideal clinical reasoning process, is therefore highly desirable.

### 4.4. Interpretability, Calibration, and Clinical Utility

Additionally, the quantitative performance of IDF-Net’s predictions was interpretable and calibrated. The Grad-CAM++ saliency maps showed strong alignment with actual lesion locations on average, with the highlighted regions overlapping with the expert-annotated tumor areas, achieving a significant performance. This confirms that the model’s visual focus aligns with the actual pathology. Likewise, SHAP analysis provided quantified contributions from each modality for each case. IDF-Net’s probability outputs were also well-calibrated; the calibration curve closely tracked the ideal diagonal, indicating that a predicted risk corresponded closely to the observed prevalence of malignancy. DCA further confirmed clinical utility, as it yielded higher significance across a wide range of threshold probabilities (0.05–0.50%) when using IDF-Net’s predictions, compared to the “*treat all*” or “*treat none*” strategies. In practical terms, this means the model can improve decision-making by reducing unnecessary interventions while increasing the detection of true cancers (a high true-positive rate) for plausible risk thresholds.

Interpretability was evaluated using two complementary metrics. First, Grad-CAM++ heatmaps were compared against expert-delineated lesion regions using the IoU metric. Second, SHAP values quantified modality-level contributions for each prediction, producing patient-specific attribution profiles. Additionally, two radiologists and two pathologists performed blinded evaluation of the interpretability outputs using a 4-point Likert scale to assess clinical plausibility. This expert validation supports IDF-Net’s potential role in augmenting physician decision-making, increasing trust in AI outputs, and improving transparency, critical factors for deployment in real clinical settings.

Overall, the integration of dynamic gating, cross-modal attention, and interpretable outputs enables IDF-Net to emulate key aspects of real clinical reasoning in CRC diagnosis. By integrating endoscopy, CT, and histopathology data, the model provides a more comprehensive and transparent decision support tool that may enhance diagnostic accuracy, reduce inter-observer variability, and streamline clinical workflows.

### 4.5. Limitations and Future Directions

The proposed model demonstrated significant performance but has some limitations. (1) The multimodal cases were constructed through diagnosis-matched pairing from separate public datasets, as naturally paired multimodal public datasets are currently unavailable. While we performed sensitivity analyses and rigorous label harmonization to mitigate this issue, latent domain shifts across imaging devices and institutional protocols may persist. This remains a key limitation of our retrospective study. (2) The retrospective design limits evaluation of IDF-Net’s performance in clinical workflows, including endoscopy and pathology review. (3) Imaging quality and acquisition parameters varied across institutions, which may introduce heterogeneity that IDF-Net partially but not fully normalizes. (4) The current framework solves a binary classification task (benign vs. malignant).

Future work will focus on extending IDF-Net’s utility by: (1) expanding its task from binary classification to detailed TNM staging, leveraging its inherent capacity to integrate structural (CT) and cellular (histopathology) information; (2) conducting prospective validation in real-world clinical pathways using both trimodal and bimodal cohorts to assess key endpoints such as adenoma detection rate and workflow efficiency; (3) exploring the regulatory pathways necessary for clinical deployment; and (4) extending the modular framework to incorporate additional data modalities, such as genomic markers or electronic health record data.

## 5. Conclusions

In this work, we introduced IDF-Net, a dynamic and interpretable cross-modal fusion framework for CRC diagnosis. By integrating endoscopy, CT, and histopathology through dual-stage gating and cross-modal attention, IDF-Net achieved superior diagnostic performance while providing quantifiable, case-specific interpretability. These findings demonstrate that dynamic multimodal fusion, combined with quantitative interpretability, can better approximate clinical decision-making than unimodal or static-fusion approaches. IDF-Net therefore represents a promising foundation for clinically reliable, transparent AI systems in gastrointestinal diagnostics. Future work will include targeted prospective validation using both trimodal and bimodal (endoscopy + histopathology) clinical cohorts to assess real-world utility. This will include evaluation against key clinical endpoints such as adenoma detection rate and diagnostic confidence, bridging model performance with tangible clinical outcomes.

## Figures and Tables

**Table 1:** Comparative analysis of IDF-Net against recent state-of-the-art multimodal AI systems for cancer diagnosis.

Model	Application	Modalities	Fusion Strategy	Key Innovation	Performance (AUC)	Interpretability
DeepFuse-CRC [3]	Lung & Colon Cancer	Histopathology	Early Concatenation	Multi-class classification	0.956	Class activation maps
GAIT [13]	GI Disease Detection	Endoscopy, Clinical	Generative AI + CNN	Synthetic data augmentation	0.931	Attention visualization
Wang et al. [23]	CRC Classification	Histopathology	ViT	Unimodal, patch-level	0.988 (patch-level)	Attention maps
M3F-Net [10]	General Medical Diagnostics	MRI, CT, Clinical	Tensor Fusion	Static fusion	0.942	Post hoc LIME
Multi-IOMM [24]	Digestive Tract Tumors	Endoscopy, Histopathology	Late Fusion	-	-	-
IDF-Net (Ours)	CRC Diagnosis	Endoscopy, CT, Histopathology	Dynamic gating + Cross-Attention	Case-specific weighting, integrated XAI	0.991 ± 0.012	Grad-CAM++, SHAP

**Table 2:** Comparative diagnostic performance of unimodal and multimodal models (mean values with 95% CI).

Model Type	Imaging Modality	Accuracy (95% CI)	Sensitivity (95% CI)	Specificity (95% CI)	F1 Score (95% CI)	AUC (95% CI)
Unimodal	CT (TCGA-COAD, ColonCancerCT)	0.897 (0.878–0.915)	0.884 (0.864–0.905)	0.908 (0.889–0.926)	0.882 (0.861–0.903)	0.911 (0.889–0.933)
	Endoscopy (HyperKvasir + Kvasir)	0.918 (0.902–0.934)	0.921 (0.906–0.937)	0.914 (0.897–0.926)	0.904 (0.886–0.921)	0.923 (0.905–0.941)
	Histopathology (LC25000, TCGA)	0.902 (0.887–0.918)	0.895 (0.877–0.912)	0.907 (0.891–0.922)	0.894 (0.874–0.912)	0.935 (0.919–0.951)
Multimodal (IDF-Net)	Endoscopy + CT + Histopathology	0.920 (0.906–0.936)	0.964 (0.953–0.976)	0.953 (0.938–0.965)	0.918 (0.901–0.933)	0.991 (0.965–0.997)

**Table 3:** External validation performance of the proposed model across modalities (with 95% CI).

Imaging Modality	Dataset	Cancer Type/Organ	Accuracy (95% CI)	F1-Score (95% CI)	AUC (95% CI)
CT (Radiology)	TCGA-COAD + ColonCancerCT	Colorectal adenocarcinoma	0.923 (0.913–0.940)	0.923 (0.912–0.939)	0.971 (0.930–0.989)
Endoscopy	HyperKvasir + GastroVision	Colorectal and Gastric cancers	0.911 (0.910–0.924)	0.912 (0.917–0.936)	0.969 (0.963–0.988)
Histopathology	LC25000 + TCGA-COAD	Colorectal adenocarcinoma	0.956 (0.932–0.964)	0.955 (0.936–0.968)	0.997 (0.958–1.000)

**Table 4:** Ablation study performance.

Model Configuration	Dynamic Routing Module (DRM)	Cross-Attention Fusion (CAF)	Interpretability Module	Accuracy (95% CI)	AUC (95% CI)
Baseline CNN (no fusion)	✗	✗	✗	0.874 (0.857–0.891)	0.923 (0.907–0.939)
+DRM only	✓	✗	✗	0.912 (0.898–0.928)	0.941 (0.926–0.957)
+DRM + CAF	✓	✓	✗	0.911 (0.894–0.928)	0.949 (0.934–0.964)
Full IDF-Net (DRM + CAF + IM)	✓	✓	✓	0.920 (0.906–0.936)	0.991 (0.965–0.997)

**Table 5:** Performance of IDF-Net under missing-modality scenarios.

Available Modalities	Available Modalities	Accuracy	AUC
Missing Endo	CT + Histo	0.904 (0.887–0.923)	0.953 (0.937–0.969)
Missing CT	Endo + Histo	0.898 (0.879–0.917)	0.948 (0.930–0.962)
Missing Histo	CT + Endo	0.885 (0.867–0.904)	0.932 (0.915–0.949)
Endoscopy + CT + Histo	All 3	0.920 (0.907–0.936)	0.991 (0.965–0.997)

IDF-Net remains robust even with missing inputs, a key innovation for real-world deployment.

## Data Availability

All datasets used in this study are publicly available from established open-access repositories. The sources are as follows: **(1) Histopathology images:** LC25000 dataset and TCGA-COAD datasets are available at https://www.kaggle.com/datasets/javaidahmadwani/lc25000?resource=download (accessed on 10 October 2025) and https://www.kaggle.com/datasets/joangibert/tcga_coad_msi_mss_jpg (accessed on 10 October 2025). **(2) Endoscopy images:** HyperKvasir, Kvasir-SEG, and GastroVision datasets are publicly available via: HyperKvasir: https://datasets.simula.no/hyper-kvasir (accessed on 22 September 2025), Kvasir-SEG: https://datasets.simula.no/kvasir-seg (accessed on 22 September 2025), and GastroVision: https://osf.io/gvx3q (accessed on 22 September 2025). **(3) Computed Tomography (CT) images:** ColonCancerCT-2025 and TCGA-COAD datasets is available through https://www.kaggle.com/datasets/orvile/coloncancerct-2025-abdominal-ct-scans (accessed on 23 September 2025) and https://www.cancerimagingarchive.net/collection/tcga-coad/ (accessed on 23 September 2025, respectively. All data were used solely for research and are fully anonymized at the source.

## Abbreviations

AI	Artificial Intelligence
CRC	Colorectal cancer
CAD	Computer-aided diagnosis
CT	Computed tomography
CI	Confidence interval
CNN	Convolutional Neural Network
CAF	Cross-attention fusion
DCA	Decision curve analysis
DL	Deep learning
DRM	Dynamic routing module
IDF-Net	Interpretable Dynamic Fusion Network
Eq	Equation
GIDs	GI diseases
Grad-CAM	Gradient-weighted class activation mapping
GPU	Graphical processing unit
QKV	Query–key–value
SHAP	SHapley Additive exPlanations
SD	Standard deviation
ViT	Vision Transformer
